# Microbiological and chemical characteristics of beaches along the Taranto Gulf (Ionian Sea, Southern Italy)

**DOI:** 10.1007/s10661-022-10103-x

**Published:** 2022-05-23

**Authors:** Osvalda De Giglio, Marcella Narracci, Francesca Apollonio, Francesco Triggiano, Maria Immacolata Acquaviva, Carmela Caroppo, Giusy Diella, Antonella Di Leo, Fabrizio Fasano, Santina Giandomenico, Lucia Spada, Rosa Anna Cavallo, Maria Teresa Montagna

**Affiliations:** 1 Department of Biomedical Science and Human Oncology, University of Bari Aldo Moro, Piazza G. Cesare 11, 70124 Bari, Italy; 2 National Research Council (CNR), Water Research Institute (IRSA), S.S. of Taranto, via Roma 3, 74123 Taranto, Italy

**Keywords:** Beachgoers, Phytoplankton, Sand, Seawater pollution

## Abstract

Coastal habitats provide important ecosystem services, such as the maintenance of ecological sustainability, water quality regulation, nutrient recycling, and sandy beaches which are important areas for recreation and tourism. The quality of seawater is generally measured by determining the concentrations of *Escherichia coli* and intestinal Enterococci, which might be affected by the persistent populations of these bacteria in sand. Sand might thus be a significant source of pathogen exposure to beachgoers. The quality of coastal recreational waters can also be affected by eutrophication, water discoloration, and harmful algal blooms, which pose additional human health risks. Here, we conducted a monitoring of the beaches quality along the Taranto Gulf by determining the concentrations of fecal indicator organisms, as well as other parameters that are not traditionally measured (physicochemical parameters, *Pseudomonas aeruginosa*, and harmful microalgae), in shallow seawater and sand sampled from three beaches. The concentrations of bacteria were determined using both standard microbiological methods and the IDEXX system. Our results demonstrate the utility of measuring a greater number of parameters in addition to those conventionally measured, as well as the importance of assessing the health risks posed by the sand matrix. Additional work is needed to develop rapid analytical techniques that could be used to monitor the microbiological parameters of solid matrices.

## Introduction

Beaches are important areas for recreation and tourism, and beachgoers generally select beaches on the basis of their perceived cleanliness. However, an ostensibly clean beach is not necessarily free of human health hazards because sand can be rich in microbes. Assessments of the microbiological quality of beach sand and seawater are thus essential for evaluating the safety of beaches for public use (Abreu et al., 2016; Botero et al., 2015; Pereira et al., 2013; WHO, 2021). The growth in beach recreation has increased the importance of monitoring various microbiological, physical, and chemical indicators of the quality of beaches in coastal management programs (Samarasekera & Abeygunawardena, 2017).

Recent studies have suggested that direct exposure to beach sand through physical contact (skin, eyes, and ears) or inhalation and ingestion is a risk factor for infectious diseases, particularly in children (Ge et al., 2012; Russell et al., 2012; Solo-Gabriele et al., 2016). Most of the microorganisms in sand, including bacteria, viruses, protozoa, helminths, and fungi, are of environmental origin, but they can also be of animal or human origin (Bonanno Ferraro et al., 2021; Montagna et al., 2010). Pathogenic bacteria that have been detected in beach sands to date include *Vibrio vulnificus*, *Salmonella*, *Campylobacter*, *Pseudomonas aeruginosa*, and *Staphylococcus aureus* (including methicillin-resistant strains) (Abdelzaher et al., 2010; Esiobu et al., 2004; Goodwin et al., 2012; Plano et al., 2013; Shah et al., 2011; Yamahara et al., 2012).

The concentration of fecal organisms in seawater is the main indicator of beach quality on which current legislation and beach management practices in Italy are based (Ministero della Salute, 2010, March 30). The concentration of fecal organisms in sand, by contrast, is not considered in current legislation. However, sand can be an environmental reservoir for pathogens (Abdelzaher et al., 2010; Sabino et al., 2014; Yamahara et al., 2012), and water and sand quality are often linked (Phillips et al., 2011; Piggot et al., 2012; Whitman et al., 2014). Generally, few studies have examined the link between fecal markers and pathogens in sand and seawater.

The quality of coastal waters is affected by the content of inorganic macronutrients (phosphate, nitrate, nitrite, and ammonium) which, coupled with sunlight and inorganic carbon, is a key factor regulating the abundance, growth, and metabolism of phytoplankton (Edler & Elbrächter, 2010). An increase in nutrient inputs can lead to eutrophication, which is measured by the concentration of chlorophyll *a* (Chl-*a*). Furthermore, the availability of nutrients affects phytoplankton biodiversity, which is an important indicator of the environmental quality of coastal ecosystems (Cerino et al., 2019; Zingone et al., 2021).

In coastal waters, changes in the color and transparency of water and the proliferation of phytoplankton can result in temporary changes in the appearance of seawater. This abnormal discoloration of seawater can affect its aesthetic appearance, which has no effect on human health unless harmful algal species are present (Figueras et al., 2015). The blooms of some harmful species that produce marine toxins are often associated with human diseases (Davidson et al., 2012). In the Mediterranean Sea in Southern Italy, most of the algal species involved in harmful blooms are planktonic, but an increase in the abundance of benthic microalgae has been observed since the 1990s (Berdalet et al., 2017; Caroppo & Bisci, 2010). *Amphidinium carterae*, *Coolia* cf. *monotis*, and *Ostreopsis* cf. *ovata* are the main components of these assemblages in the Northern Ionian Sea, a marine basin that occupies the westernmost portion of the eastern Mediterranean Sea (Pagliara & Caroppo, 2012).

Here, we employed a new approach to monitoring the quality of sea water and sand in the Gulf of Taranto (Ionian Sea). Specifically, we evaluated various microbiological, phytoplankton, and physicochemical parameters of seawater, including conventional (*Escherichia coli* and Enterococci) and unconventional (*Pseudomonas aeruginosa*) microbiological parameters using both traditional and novel methods (in reference to IDEXX system for *P.aeruginosa* detection). Simultaneously, the same microbiological parameters were investigated in the sand with traditional and innovative methods (in reference to IDEXX system for *E.coli*, Enterococci and *P.aeruginosa*).

## Materials and methods

### Study area

The Mediterranean Sea is a semi-closed and shallow sea connected to the Atlantic Ocean through the Strait of Gibraltar. The southern coast of Italy is surrounded by the Tyrrhenian Sea, the Adriatic Sea, and the Ionian Sea, the latter of which is the deepest Italian sea (exceeding 4,000 m in some areas). In the winter, the temperature of the surface water and immediately below the surface is approximately 13 °C; in the summer, the temperature is approximately 28 °C. The salinity (38‰) varies little throughout the year.

The Gulf of Taranto, which occurs within the Ionian Sea, is the largest gulf in Italy (140 km long and 111 km wide). It is enclosed by three Italian regions, Puglia, Basilicata, and Calabria, and is made up of two basins, the Mar Grande and the Mar Piccolo. The Mar Grande is a semi-enclosed deep sea (maximum depth of 35 m and area of 36 km^2^) subject to lateral water exchange with the Ionian Sea. It is connected to the Mar Piccolo through two channels and borders the mainland, the historic center of Taranto, the two Cheradi Islands, and two artificial cliffs. The most important base of the Italian Navy is located in Mar Grande. Commercial activities, industry, and tourism are prevalent, and mussel farms are widespread. Mar Grande is thus a vulnerable and sensitive area. Three sandy beaches (St1, St2, and St3) overlooking Mar Grande were sampled in this study.

### Sample collection

**Fig. 1:**
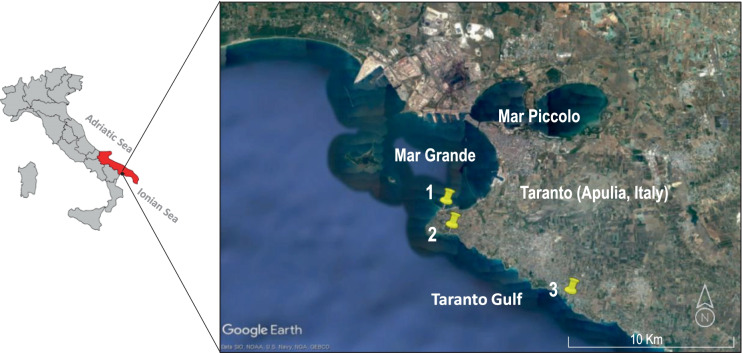
Map of the sampling locations in the Taranto Gulf, Ionian Sea, Southern Italy

The seawater of the three beaches (Fig. 1) was monitored by measuring microbiological parameters (*Escherichia coli* and Enterococci) per Italian legislation (Ministero della Salute, 2010, March 30). *Pseudomonas aeruginosa,* phytoplankton, and some physicochemical parameters were also monitored. The sand was monitored by measuring the same microbiological parameters. From April to September 2019, samples were collected monthly between 8:00 and 11:00 am on dry, calm days, transported in a refrigerator at 4 °C, and processed within 5 h. A total of 36 samples (18 seawater and 18 sand samples) were collected.

Samples of seawater (1 L) were taken approximately 2.5 m from the beach line and approximately 30 cm below the sea surface using a telescopic rod (extendable sampler for water sampling with interchangeable containers and sample holders). The water samples were collected using sterile polyethylene bottles.

The dry sand samples (50 g) were collected under aseptic conditions (using sterile gloves and in a sterile container) at approximately 2 m from the sea.

### Microbiological parameters

Water and sand samples were tested for *E. coli*, Enterococci, and *Pseudomonas aeruginosa* using two different methods: the culture-based method and the IDEXX system.

### Culture-based method

Specific aliquots of each seawater sample were filtered through a cellulose ester membrane with a diameter of 47 mm and a pore size of 0.45 µm (Millipore, Milan, Italy).

For *E. coli* and coliform bacteria, 100 mL of seawater was filtered, and the membrane was placed on plates containing Chromogenic Coliform Agar (Biolife Italiana Srl, Milan, Italy). After incubation at 36 ± 2 °C for 24 ± 2 h, the blue-violet colonies were identified as *E. coli*, and the salmon pink, oxidase-negative colonies were identified as coliform bacteria (UNI, 2017).

For Enterococci, 100 mL of seawater was filtered, and the membrane was placed on Slanetz and Bartley agar medium (Biolife Italiana Srl, Milan, Italy) and incubated at 36 ± 1 °C for 48 h. When dark pink-red colonies developed, the membrane was transferred to a plate containing Bile Esculin Azide agar (Biolife Italiana Srl, Milan, Italy) and incubated at 44 °C for 2 h. Brown colonies with brown-black halos and positive catalysis were identified as Enterococci (UNI, 2003).

For *P. aeruginosa*, 250 mL of seawater was filtered, and the membrane was placed on a plate containing Pseudomonas Selective Agar supplemented with cetrimide (0.20 g) and nalidixic acid (15 mg) (Microbiol, Cagliari, Italy) and incubated at 36 ± 2 °C for 44 ± 4 h. Blue-green pyocyanin-producing colonies were identified as *P. aeruginosa* (UNI, 2008).

Concentrations of bacteria in the seawater samples were considered compliant with the standards of the Italian Ministerial Decree (Ministero della Salute, 2010, March 30) when the concentration of *E. coli* was below 500 colony forming units (CFU)/100 mL (limit of detection, LOD < 1 CFU/100 mL) and the concentration of Enterococci was below 200 CFU/100 mL (LOD < 1 CFU/100 mL).

Regarding *P. aeruginosa*, the seawater was considered suitable for bathing when the microorganism was absent in 250 mL (LOD < 1 CFU/250 mL).

Each sand sample (50 g) was transferred to Pyrex glass bottles containing 450 mL of sterilized distilled water. A sterile magnetic stirrer was added, and vigorous stirring (500 rpm) was performed for approximately 30 min to resuspend the bacteria. One-mL aliquots of the supernatant produced after sedimentation were seeded on TBX agar for *E. coli* detection (incubation at 44 °C for 18–24 h), Slanetz and Bartley agar for Enterococci detection (36 °C ± 1 for 44 ± 4 h), and Pseudomonas Selective Agar supplemented with cetrimide (0.20 g) and nalidixic acid (15 mg) for *P. aeruginosa* detection (36 °C ± 1 for 44 ± 4 h). The results were expressed in CFU/g of sand, and the detection limit was < 10 CFU/g.

### IDEXX system

*E. coli*, Enterococci, and *P. aeruginosa* in seawater and sand samples were detected using the Colilert, Enterolert, and Pseudalert test kits (IDEXX Laboratories, Maine, USA), respectively.

To detect *E. coli*, 10 mL of seawater was added to 90 mL of sterile distilled water. Colilert dehydrated culture medium (IDEXX) was then added to the solution, left to stand for 5 min, and homogenized. The solution was then poured into a Quantitray (IDEXX), sealed in an appropriate device (IDEXX), and placed in an incubator at 35 ± 0.5 °C for 18–22 h. The same procedure was used to detect Enterococci, except that Enterolert (IDEXX) was used as a culture medium and the solution was kept in an incubator at 41 ± 0.5 °C for 24–28 h.

To detect *P. aeruginosa*, Pseudalert (IDEXX) was used as the culture medium, and samples were incubated at 38 ± 0.5 °C.

Sand samples were analyzed using the same procedure. Under aseptic conditions, 50 g of each sample was weighed and transferred to Pyrex glass flasks containing 450 mL of sterile distilled water. A sterile magnetic stirrer was added, and vigorous stirring was performed for approximately 30 min to resuspend the bacteria (Abreu et al., 2016; Pereira et al., 2013). 10 mL of the supernatant was extracted into a sterile vessel (IDEXX) and filled with sterilized distilled water to a final volume of 100 mL. Next, dehydrated culture media were added to the solution, Colilert (IDEXX) for *E. coli*, Enterolert (IDEXX) for Enterococci, and Pseudalert (IDEXX) for *P. aeruginosa*; the solutions were then placed in the incubator at their respective temperatures.

After incubation, wells positive for *E. coli*, Enterococci, and *P. aeruginosa* were counted under ultraviolet light (λ = 360 nm). The bacterial count was quantified using an MPN table, and the results were expressed in MPN/100 mL for water and MPN/g for sand. The seawater samples were considered compliant with the standards of the Italian Ministerial Decree (Ministero della Salute, 2010, March 30) when the concentration of *E. coli* was below 500 MPN/100 mL and the concentration of Enterococci was below 200 MPN/100 mL. Regarding *P. aeruginosa*, the sand was considered suitable for bathing when the microorganism was absent in 10 g (LOD < 10 CFU/g).

### Phytoplankton counts and microalgae identification

Recently collected seawater samples were immediately fixed with Lugol's iodine solution and examined using an inverted microscope (Labovert FS Leitz) equipped with a phase-contrast objective at magnifications of 400 × and 630 × . Subsamples varying from 50 to 100 mL were allowed to settle for 24–48 h depending on the density of phytoplankton, and phytoplankton densities were determined using the Utermöhl method (Edler & Elbrächter, 2010). Counts were performed along transects (1–4) or in random fields (30–60); in addition, half of the Utermöhl chamber was examined at a magnification of 200 × to ensure that less abundant microalgal taxa were counted.

### Physicochemical parameters

Several physicochemical parameters were measured for each seawater sample. Temperature and pH were recorded in situ by a digital thermometer with a stainless steel sensor probe and a digital pH meter model HD8602 (Beckman Coulter^®^, Milan, Italy), respectively. Dissolved oxygen (DO) was measured by Winkler's titration method (Lundholm et al., 2009) through the oxidation of Mn^2+^ to MnO_2_ under basic conditions. Salinity was determined using a CDM 83 conductivity meter (Radiometer Copenhagen, Denmark) within 4 h after sampling.

The content of nitrite (N-NO_2_), ammonium (N-NH_4_), and ortho-phosphates (P-PO_4_) was determined using a multi-nutrient auto-analyzer system (EasyChem Plus, Systea SpA, Anagni, Italy), which is a modular multiparametric analyzer for the automatic monitoring of nutrients in seawater that employs the wet-chemical colorimetric analysis method (Ministero Ambiente e Tutela del Territorio, 2001). Analysis of the nitrate (N-NO_3_) content was conducted using automatic UV digestion (mod. μMAC-1000 of Systea SpA, Anagni, Italy) with DTPA (diethylene triamine pentacetic) to reduce NO_3_ to NO_2_. Finally, the nitrate–N content was calculated by subtracting the nitrite concentration from the NO_3_ concentration.

The water samples for the analysis of Chl-*a* were stored in the dark and kept at 4 °C until filtration. Chl-*a* was measured fluorimetrically with an RF-1501 fluorescence spectrofluorimeter (Shimatzu, Milan, Italy) following the Environmental Protection Agency method 445.0 (Arar & Collins, 1997). Specifically, 250 mL of seawater was filtered through a Whatman GF/F 47 Ø mm filter under vacuum at low pressure; subsequently, the filters were extracted with an acetone/water mixture (90% v/v). Before quantification, the extracts were centrifuged, and the concentration of Chl-*a* was determined before and after acidification with HCl. All analyses were conducted in triplicate.

## Statistical analyses

Quantitative data for the microbiological analysis were presented as the median and interquartile range because Gaussian distributions could not be assumed. A descriptive analysis was performed on the microbial and physicochemical parameters of the water and sand samples from each beach. Statistical analyses were carried out to verify the consistency between estimates of MPN/100 mL and CFU/100 mL for water and MPN/100 mg and CFU/g for sand samples across all beach samples (presence/absence) using the McNemar test. Because the limits of MPN/100 mL and CFU/100 mL for water were 10 and 1, respectively, the methods were considered consistent when the values of MPN/100 mL and CFU/100 mL were < 10 and < 1–9, respectively, and when MPN/100 mL and CFU/100 mL both had values of > 10. Because the limit of MPN/g and CFU/g for sand was 10, the methods were considered consistent when MPN/g and CFU/g both had values of < 10 or > 10. Differences in the degree of contamination of the water and sand samples at all beaches determined via the standard culture-based method were evaluated using Wilcoxon signed-rank tests with continuity correction tests. Kruskal–Wallis chi-squared was used to compare the values ​​of different microorganisms between stations. The effects of physicochemical parameters on variation in *E. coli*, *P. aeruginosa*, and Enterococci in seawater samples were evaluated using Poisson regression models. R software (version 4.0) was used to conduct all statistical analyses, and the threshold for statistical significance for all tests was p < 0.05.

## Results and discussion

### Microbiological parameters

#### Water

Italy has the largest number of bathing establishments (representing approximately a quarter of all European bathing areas) among all European countries, including 11,000 privately managed “bathing establishments,” and has more than 50,000 state-owned maritime concessions (). Given that beaches are highly popular tourist destinations, the environmental quality of beaches is an important factor affecting the attractiveness of beaches to tourists (Pereira et al., 2013). No statistically significant differences in the degree of microbial contamination of the water were observed among the beaches sampled in this study (*E. coli*—Kruskal–Wallis chi-squared = 0.8856, *p-value* = 0.64; *Enterococci*—Kruskal–Wallis chi-squared = 3.7584, *p-value* = 0.15; *P. aeruginosa*—Kruskal–Wallis chi-squared = 2.4034, *p-value* = 0.30) (Table 1). The concentrations of *E. coli* and Enterococci in the waters were lower than the limits stipulated by Italian legislation (200 CFU or MPN/100 mL for Enterococci; 500 CFU or MPN/100 mL for *E. coli*) (Ministero della Salute, 2010, March 30), which indicates that the waters are suitable for bathing. However, a low level of *E. coli* and Enterococci contamination was observed in all three beaches in the summer months (June and July). *P. aeruginosa* was only detected on one beach (St1) in August. It is a free-living bacterium that mainly occurs in soil, seawater, and natural waters (lakes and rivers); it is one of the most important opportunistic pathogens of humans and can cause eye and skin diseases, pneumonia, and sepsis. Its infections are often severe and associated with high mortality, especially if the strains are multi-drug resistant (Ghorpade et al., 2019). Therefore, the presence of *P. aeruginosa* in water used for recreational use poses a public health problem.

**Table 1:** Microbiological contamination of water samples from three beaches along the Taranto Gulf from April to September 2019

Beaches	St1		St2		St3	
	Median value (range)		Median value (range)		Median value (range)	
Microorganisms	MPN/100 mL	CFU/100 mL	MPN/100 mL	CFU/100 mL	MPN/100 mL	CFU/100 mL
*E. coli*	5 (< 10–20)	10 (2–29)	10 (< 10–242)	7 (< 1–60)	< 10	6 (3–29)
Enterococci	10 (< 10–52)	6 (< 1–19)	< 10	< 1	< 10	1 (< 1–6)
*P. aeruginosa*	< 10	< 1	< 10 (< 10–41)	< 1 (< 1–13)	< 10	< 1 (< 1–8)

Comparison of the MPN/100 mL and CFU/100 mL values for *E. coli* (McNemar, p = 1)*,* Enterococci (McNemar, p = 0.25), and *P. aeruginosa* (McNemar, p = 1) of the water samples of all three beaches revealed that there were no statistically significant differences in the level of microbial contamination estimated using the two methods. The complete concordance of the CFU/100 mL and MPN/100 mL data confirms that these methods are equally effective and validated for monitoring the microbiological parameters of seawater samples.

#### Beach sand

The microbiological quality of bathing water is one of the measures currently used to evaluate the environmental and sanitary quality of beaches. However, the sands of the coastal zone also provide a habitat suitable for microorganisms (Halliday & Gast, 2011) as Enterococci (quantitative value over 1000 MPN/g) and *E.coli* (quantitative value lower of Enterococci) (Abdelzaher et al., 2010; Shah et al., 2011). Regardless of the source of contamination, the incidence of infection is higher among beachgoers compared with non-bathers and higher among bathers who normally play in the sand compared with those who do not (Fleisher et al., 2010; Heaney et al., 2012). Therefore, the microbiological monitoring of beach sand is critically important for robustly evaluating the safety of beaches for public use. Studies of beach sand quality and monitoring are becoming increasingly important, given the increase in public awareness of the potential health hazards of beaches and the increase in the popularity of beaches as holiday tourist destinations (Sabino et al., 2014). Table 2 shows the median level of microbiological contamination of the sand samples at each beach.

**Table 2:** Microbiological contamination of sand samples from three beaches along the Taranto Gulf from April to September 2019

Beaches	St1		St2		St3	
	Median value (range)		Median value (range)		Median value (range)	
Microorganisms	MPN/g	CFU/g	MPN/g	CFU/g	MPN/g	CFU/g
*E. coli*	< 10	< 10	< 10 (< 10–2,142)	< 10 (< 10–24)	< 10 (< 10–41)	< 10
Enterococci	1995 (10–6070)	13 (< 10–1,660)	186 (< 10–738)	1 (< 10–16)	5 (< 10–1,396)	< 10
*P. aeruginosa*	< 10 (< 10–101,120)	< 10 (< 10–12,160)	< 10 (< 10–369)	< 10 (< 10–10)	< 10 (< 10–631)	< 10

The only difference observed among beaches according to the culture-based method (CFU/g) was in Enterococci, which was higher at St1 than at St2 and St3 in August. The concentration of Enterococci (CFU/g) in samples was almost always higher than that of *E. coli*. This is consistent with the greater environmental resistance of Enterococci relative to *E. coli*, which is more susceptible to desiccation and solar radiation. Therefore, according to recent “Guidelines on recreational water quality” (WHO, 2021), our results indicate that Enterococci provides a more suitable indicator for assessing the level of fecal contamination (Halliday & Gast, 2011) and that *E. coli* should not be used as the sole bacterial indicator of sand quality (Locas et al., 2008). More precisely, the new WHO guidelines recommend only Enterococci and not *E.coli* for sand monitoring with a provisional limit of 60 MPN or CFU/g based on the Quantitative Microbial Risk Assessment (QMRA) used to indirectly estimate the risk to human health (WHO, 2021). As some of our sand results are clearly above this limit value, several efforts should focus on preventive measures to reduce the risk of sand contamination by Enterococci. For example, it is necessary to limit access to the beach by dogs and wild animals, such as cats, and to prepare management plans for birds. In addition, properly designed solid waste disposal facilities, adequate sewage and sludge treatment and stormwater drainage should be provided (WHO, 2021). According to other authors (Esiobu et al., 2004; Tugrul-Icemer & Topaloglu, 2011), *P. aeruginosa* was also been detected in beach sand in higher concentrations at St1 than at St2 and St3.

Generally, parents should ensure that their children engage in safe behaviors and prevent them from coming into direct contact with sand for long periods. Children should also be instructed to not put their hands in their mouth after playing in the sand (Montagna et al., 2020a). Although the utility of the IDEXX method for detecting the level of microbiological contamination of seawater has been previously confirmed, researchers that have used this approach on sand (Pereira et al., 2013) have not compared its efficacy with that of standard culture-based methods. The results of our study indicated that the MPN/g and CFU/g estimates were concordant for all microorganisms, with the exception of Enterococci.

There were no significant differences in the MPN/g and CFU/g values for *E. coli* between the two methods (McNemar, *p* = 0.62), and the values exhibited a concordance of 77.8%. Values of MPN/g and CFU/g for Enterococci significantly differed between the two methods (McNemar, *p* = 0.03), which exhibited a concordance of 44.4%. Values of MPN/g and CFU/g for *P. aeruginosa* did not significantly differ between the two methods (McNemar, *p* = 0.13) and exhibited a concordance of 77.8%. In all cases when discrepancies were observed, the number of positive results was higher for the MPN method than for the CFU method. Additional research with a larger number of samples is needed to validate the efficacy of the IDEXX method for determining the level of microbiological contamination in matrices other than water. The IDEXX method greatly reduces the time required for analyses compared with standard culture-based methods.

#### Comparison of the level of microbiological contamination of water and beach sand according to the culture-based method

Seawater is a potential source of contamination. Phillips et al. (2011) characterized the relationship between average beach sand and seawater quality. We found that the concentration of *E. coli* was significantly higher in water than in sand according to the culture-based method (Wilcoxon signed-rank test with continuity correction test: V = 114, *p* = 0.002). However, no statistically significant differences were observed between water and sand for Enterococci (V = 29, *p* = 0.262) and *P. aeruginosa* (V = 10, *p* = 0.589).

This finding is in contrast to other studies reporting that the concentrations of fecal indicators (*E. coli* and Enterococci) are higher in sand (both dry and wet) compared with the adjacent seawater (Beversdorf et al., 2007; Hartz et al., 2008). Bacteria have a higher growth potential in sand because of the ease with which bacteria can adsorb to sand particles and organic matter; sand sediments also provide protection from solar radiation (Pereira et al., 2013; Zhang et al., 2015).

No differences in the level of microbiological contamination of the water and sand were observed among the three beaches. Beaches (namely, the water and sand) can become polluted by fecal matter through human activities, such as the deterioration or destruction of sewer lines; the ineffective treatment of wastewater; direct contact of the sand with polluted water through the tide, waves, and wind; the decay of garbage and debris left on the beach or released by ships; and animal excrement (e.g., dogs, seabirds, rodents, and humans) (Pereira et al., 2013).

### Phytoplankton and harmful microalgae

Phytoplankton abundances ranged from 72.9 × 10^3^ (Station 2, May 2019) to 60.2 × 10^4^ cells/L (Station 1, July 2019); average values were similar among all beaches, but slightly higher values were observed for St1. The community was mainly composed of undetermined phytoflagellates (range: 5.1–70.4% of the total community) and dinoflagellates (range: 7.2–85.6%). Diatoms (range: 6.1–45.1%) were more abundant at St1, and abundances of coccolithophorids (up to 5.4%) were similar among all the sampled stations.

Harmful microalgae identified in all the beaches included three diatoms (*Pseudo-nitzschia delicatissima* group, *P.* cf. *galaxiae*, and *P. pseudo-delicatissima* complex) and five dinoflagellates (*Alexandrium minutum* group, *Margalefidinium polykrikoides, Ostreopsis* cf. *ovata, Prorocentrum lima, P. cordatum,* and *P.* cf. *mexicanum*) (Lundholm et al., 2009).

Phytoplankton abundances were typical of the spring–summer communities of the coastal waters (e.g., Cerino et al., 2019). The species comprising these communities, including the harmful species, have been identified in Mediterranean waters (Zingone et al., 2021) and along the Ionian coasts (Caroppo et al., 2016). These species, which are potential producers of toxins, did not reach high concentrations and were not the cause of intoxication during our sampling period. However, the density of *Margalefidinium polykrikoides* was high (up to 66.3 × 10^3^ cells/L) at St3 in September*.* This dinoflagellate was recently responsible for a bloom that colored the waters and heavily affected the tourism of a marine protected area in the Apulia Region (Porto Cesareo) (Roselli et al., 2020), which is located close (about 60 km) to the beaches sampled in our study. Our data confirm that this species has spread along the Ionian coasts and highlight the need for improved risk assessments to prevent the occurrence of harmful blooms. Species that have occurred in low concentrations in phytoplankton assemblages in the past but have now become one of the main components of harmful blooms posing human health risks and inducing socio-economic damage merit special attention.

### Physicochemical parameters

Seasonal variation in the surface water temperature (range 17.1–27.5 °C) throughout the study period (April–September 2019) was similar among sites (Table 3). The highest and minimum values were observed in August (27.5 °C) and September (17.1 °C) at St1, respectively. The salinity at all beaches ranged from 37.3 to 38.9‰. From April to September, mean salinity values at St1 and St2 were similar (38.5 and 38.6‰, respectively); the mean value was approximately 1‰ lower at St3 (37.6‰). The pH was similar among all beaches, and the average value ranged from 7.96 to 7.98. Dissolved oxygen (DO) values ranged from 5.82 mg/l (August, St2) to 7.41 mg/l (April, St1). The oxygen saturation level was always greater than 87%, indicating that the oxygenation conditions of the surficial water from April to August were adequate.

**Table 3:** Summary of the physical–chemical parameters of the marine water samples at St1, St2, and St3 from April to September 2019

**Year 2019**	**T**	**pH**	**DO**	**DO**	**SAL**	**P (PO**_**4**_**)**	**N (NH**_**4**_**)**	**N (NO**_**2**_**)**	**N (NO**_**3**_**)**	**Chl-*a***
	**(°C)**		**(mg/L)**	**(% sat.)**	**(**‰**)**	**(µg/L)**	**(µg/L)**	**(µg/L)**	**(µg/L)**	**(µg/L)**
	**St1**									
**April**	17.1	7.98	7.41	97.1	38.8	7.3	10.5	< 1.5	< 9.0	1.2
**May**	19.1	8.00	6.50	88.1	38.1	4.0	16.5	< 1.5	< 9.0	1.9
**June**	25.0	7.79	6.00	90.2	38.3	4.5	25.3	< 1.5	< 9.0	1.4
**July**	26.7	8.32	6.50	100	38.1	2.0	36.0	2.9	12	0.9
**August**	27.5	8.14	6.30	99.1	38.8	2.0	29.3	< 1.5	< 9.0	0.4
**September**	24.6	7.59	6.00	90.0	38.8	3.0	71.7	1.9	< 9.0	0.5
***Mean***	23.3	7.97	6.45	94.2	38.5	3.8	31.5	1.8	9.5	1.1
	**St2**									
**April**	19.3	7.90	7.20	98.3	38.9	4.6	8.4	< 1.5	< 9.0	0.9
**May**	20.1	7.92	6.30	86.9	38.1	3.1	20.7	1.9	12	0.2
**June**	24.2	7.95	6.40	95.0	38.1	2.7	15.7	< 1.5	< 9.0	0.2
**July**	25.1	8.16	6.95	104	38.8	8.0	24.1	2.9	< 9.0	0.5
**August**	25.0	7.95	5.82	88.0	38.8	6.7	19.1	< 1.5	< 9.0	0.9
**September**	25.6	7.86	6.20	94.5	38.8	7.6	30.5	3.0	< 9.0	0.5
***Mean***	23.2	7.96	6.48	94.5	38.6	3.8	19.8	2.1	9.5	0.5
	**St3**									
**April**	19.4	8.00	7.40	101	37.9	3.5	8.6	< 1.5	177	0.6
**May**	18.2	7.95	7.10	94.4	37.9	2.4	13.7	3.4	194	0.3
**June**	23.5	7.98	7.05	103	37.3	2.0	18.1	2.5	225	0.6
**July**	25.1	8.01	6.70	101	37.5	2.0	16.4	3.9	194	0.6
**August**	25.2	8.08	6.30	95.0	37.3	2.0	21.0	2.2	221	0.6
**September**	25.6	7.84	6.23	94.5	37.5	5.3	36.4	3.3	174	0.4
***Mean***	22.8	7.98	6.80	98.0	37.6	2.9	19.0	2.8	198	0.5

The data represent the average of triplicate measurements (n = 3). The standard deviations range from 3 to 9%

*DO* dissolved oxygen, *sat* saturation, *SAL* salinity, *Chl*-*a* chlorophyll *a*

The highest values of ammonium (N-NH_4_), especially at St1 (71.7 μg/L), were observed in September; values of ammonium were lower in April and ranged from 8.4 to 10.5 μg/L. High ammonium values are possibly associated with the decomposition of organic material, especially in the summer, coupled with the increase in the water temperature. Nitrite (N-NO_2_) and *ortho*-phosphate (P-PO_4_) concentrations were low at all beaches throughout the study period and ranged from n.d. to 3.9 μg/L and from n.d. to 8.0 μg/L, respectively. Nitrate (N-NO_3_) levels were low at St1 and St2 (range n.d.–12.0 μg/L); at St3, the concentration of N-NO_3_ ranged from 174 μg/L (September) to 225 μg/L (June).

The lower salinity and higher content of nitrate at St3 compared with St1 and St2 might be associated with the presence of freshwater springs along some stretches of this coastline. The presence of these freshwater springs and protection from the wind permitted the existence of an ancient city (Satyrion) from the Neolithic to the high Middle Age near St3, which was one of the most significant sites in the Mediterranean (De Luca & Fabrizio, 2016). The water wells quality in this area frequently visited by tourists is influenced by intense anthropogenic and agricultural activity on the soil. This could explain the presence of a greater contamination of nitrate in the groundwater reaching the sea water (Montagna et al., 2020b).

The content of Chl-*a* ranged from 0.2 to 1.9 µg/L across all beaches sampled. The minimum value was observed at St2 (May and June), and the maximum value was observed at St1 (May). The mean Chl-*a* values were similar to values reported by other studies for the Taranto Gulf (Alabiso et al., 2013; Caroppo et al., 2006). The content of Chl-*a* (Chl-*a* < 2.5 µg/L), a trophic state indicator, revealed that all of the coastal waters sampled were oligotrophic (Hakanson & Bryhn, 2008).

#### Effect of physicochemical factors on the microbiological parameters in water samples

Physicochemical factors had no significant effect on the concentrations of Enterococci and *E. coli* according to Poisson regression models. DO and NO_3_ were inversely and directly related to concentrations of *P. aeruginosa* (Table 4), which can grow anoxically using nitrate (Line et al., 2014).

**Table 4:** Results of the Poisson regression model for the effect of chemical parameters on the concentration of *P. aeruginosa* in water samples

	Estimate	Std. Error	t-value	*p*-value
Intercept	-243.83	128.62	-1.90	0.09
Temp	-0.46	0.42	-1.09	0.31
pH	12.32	7.20	1.71	0.13
DO	-9.08	2.78	-3.27	0.01*
SAL	5.59	3.21	1.74	0.12
PO_4_	0.50	0.50	1.00	0.35
NH_4_	-0.03	0.09	-0.36	0.73
NO_2_	-0.90	0.76	-1.19	0.27
NO_3_	0.05	0.02	2.89	0.02*
Chl-*a*	0.02	2.02	0.01	0.99

*DO* dissolved oxygen, *sat* saturation, *SAL* salinity, *Chl*-*a* chlorophyll *a*

*statistically significant

## Conclusions

The results of this study demonstrate the utility of measuring microbial parameters other than the concentrations of fecal bacteria in seawater to monitor the safety of beaches for recreational use; the efficacy of using the IDEXX system to measure microbiological parameters, which permits results to be obtained more quickly; and the importance of evaluating the level of microbiological contamination of sand for a more robust and comprehensive assessment of the public health risks of beaches. Although disinfection of sand is not recommended to reduce development of microorganisms because of negative impacts on native flora and fauna, alternative simpler methods, such as sifting and aeration, could be applied. Moreover, management and communication strategies for safety beaches include proper design of solid waste disposal facilities, provision of toilet facilities and appropriate stormwater drainage. Comprehensive sanitary surveys, coupled with visitor education, and well-designed monitoring programs, are needed to ensure that recreational beaches can be safely enjoyed by beachgoers. Strict compliance with preventative hygienic and sanitary procedures and common sense on the part of beachgoers will also help safeguard ecologically important coastal areas.

## Data Availability

All data generated or analyzed during this study are included in this published article.
